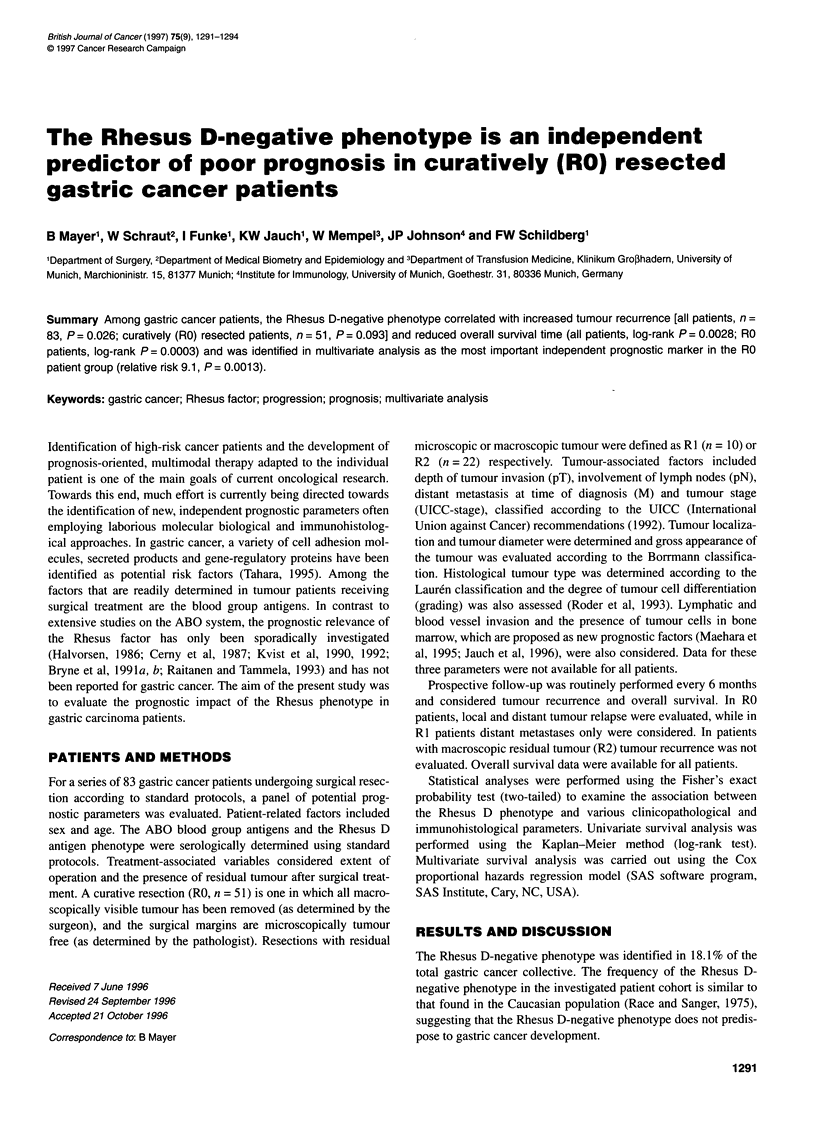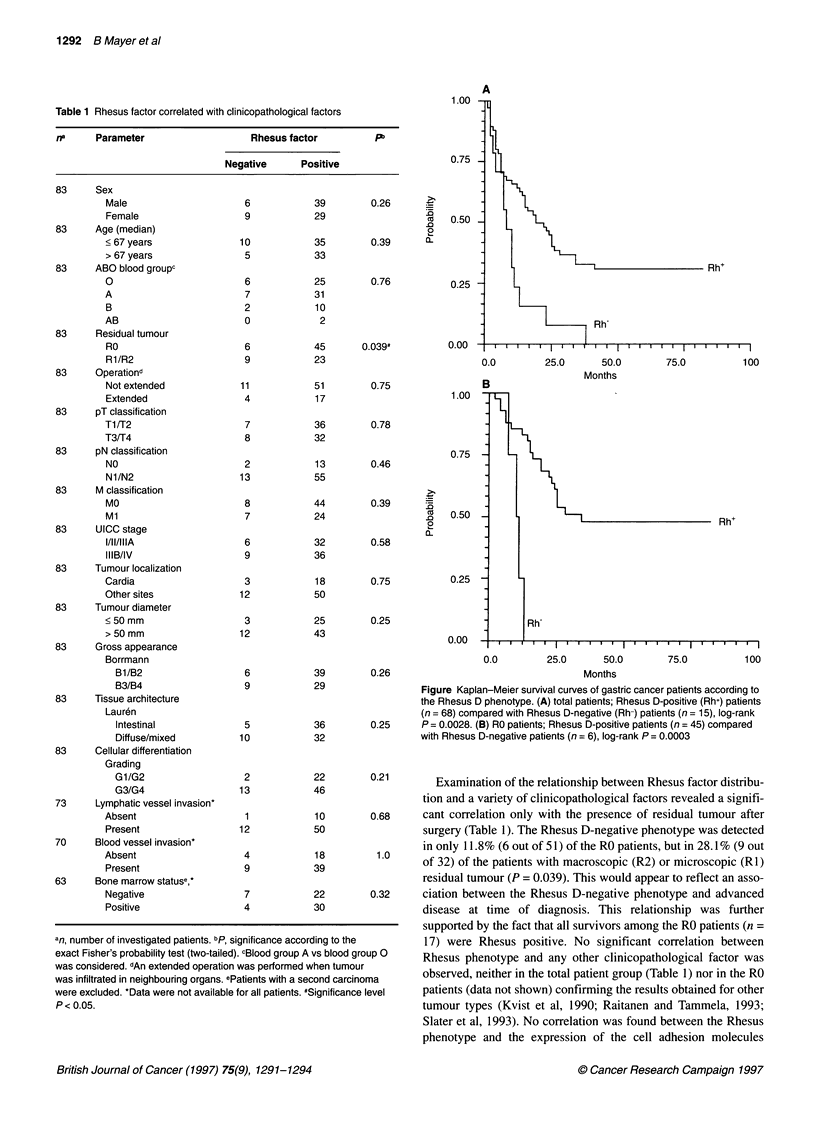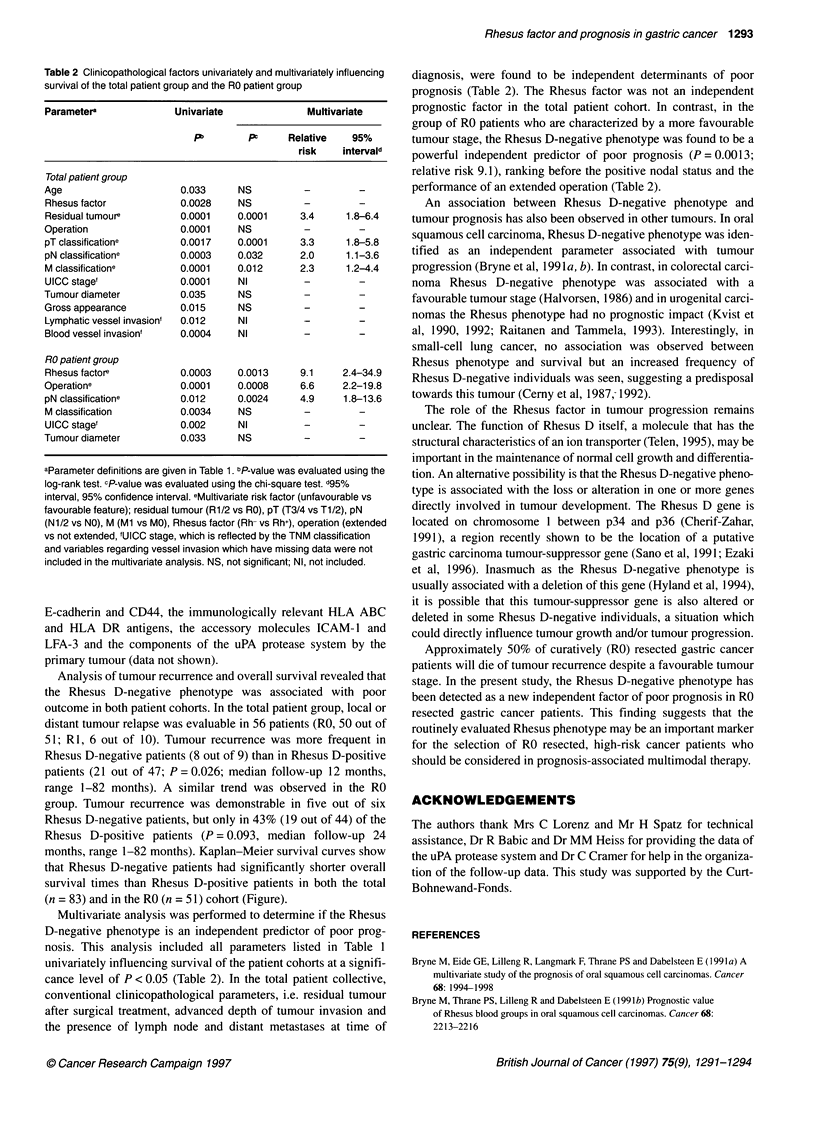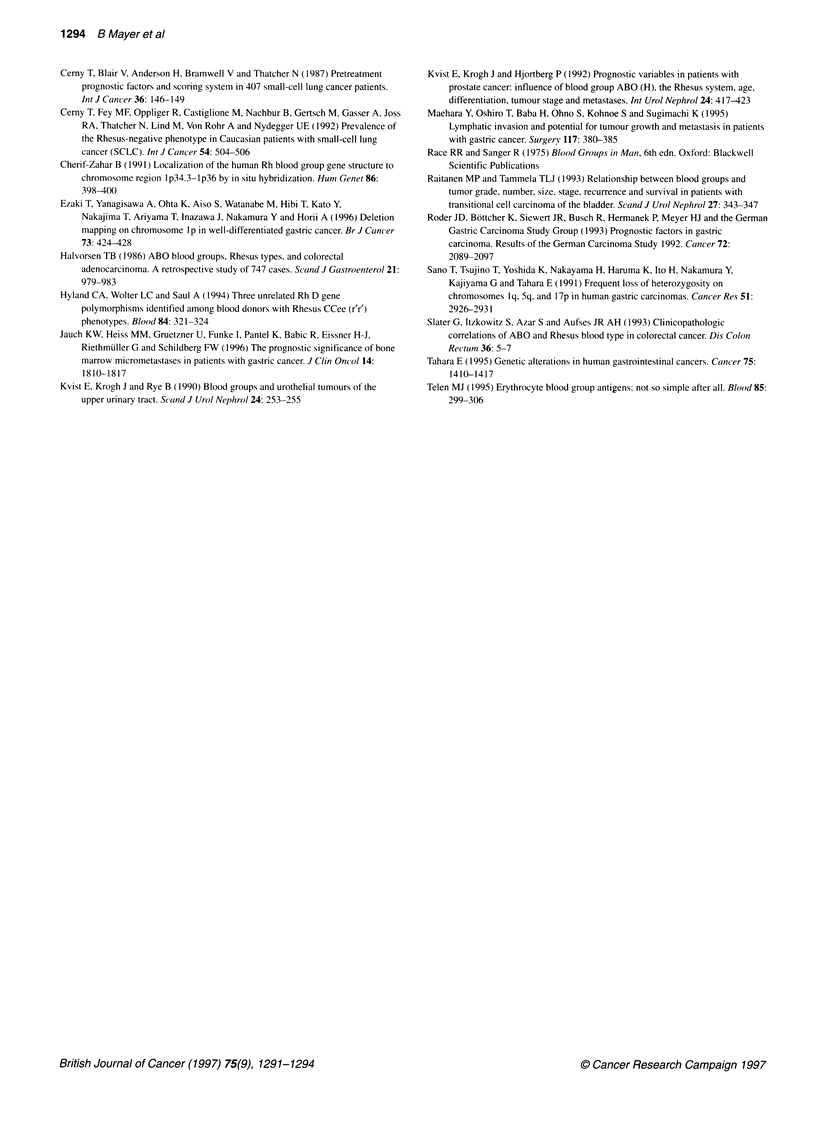# The Rhesus D-negative phenotype is an independent predictor of poor prognosis in curatively (RO) resected gastric cancer patients.

**DOI:** 10.1038/bjc.1997.219

**Published:** 1997

**Authors:** B. Mayer, W. Schraut, I. Funke, K. W. Jauch, W. Mempel, J. P. Johnson, F. W. Schildberg

**Affiliations:** Department of Surgery, University of Munich, Germany.

## Abstract

Among gastric cancer patients, the Rhesus D-negative phenotype correlated with increased tumour recurrence [all patients, n = 83, P = 0.026; curatively (R0) resected patients, n = 51, P = 0.093] and reduced overall survival time (all patients, log-rank P = 0.0028; R0 patients, log-rank P = 0.0003) and was identified in multivariate analysis as the most important independent prognostic marker in the R0 patient group (relative risk 9.1, P = 0.0013).


British Journal of Cancer (1997) 75(9), 1291–1294
© 1997 Cancer Research Campaign

### The Rhesus D-negative phenotype is an independent predictor of poor prognosis in curatively (R0) resected gastric cancer patients

**B Mayer[1], W Schraut[2], I Funke[1], KW Jauch[1], W Mempel[3], JP Johnson[4] and FW Schildberg[1]**

[1]Department of Surgery, [2]Department of Medical Biometry and Epidemiology and [3]Department of Transfusion Medicine, Klinikum Groβhadern, University of Munich, Marchioninistr. 15, 81377 Munich; [4]Institute for Immunology, University of Munich, Goethestr. 31, 80336 Munich, Germany

**Summary** Among gastric cancer patients, the Rhesus D-negative phenotype correlated with increased tumour recurrence [all patients, $n$ = 83, $P$ = 0.026; curatively (R0) resected patients, $n$ = 51, $P$ = 0.093] and reduced overall survival time (all patients, log-rank $P$ = 0.0028; R0 patients, log-rank $P$ = 0.0003) and was identified in multivariate analysis as the most important independent prognostic marker in the R0 patient group (relative risk 9.1, $P$ = 0.0013).

**Keywords:** gastric cancer; Rhesus factor; progression; prognosis; multivariate analysis

Identification of high-risk cancer patients and the development of prognosis-oriented, multimodal therapy adapted to the individual patient is one of the main goals of current oncological research. Towards this end, much effort is currently being directed towards the identification of new, independent prognostic parameters often employing laborious molecular biological and immunohistological approaches. In gastric cancer, a variety of cell adhesion molecules, secreted products and gene-regulatory proteins have been identified as potential risk factors (Tahara, 1995). Among the factors that are readily determined in tumour patients receiving surgical treatment are the blood group antigens. In contrast to extensive studies on the ABO system, the prognostic relevance of the Rhesus factor has only been sporadically investigated (Halvorsen, 1986; Cerny et al, 1987; Kvist et al, 1990, 1992; Bryne et al, 1991*a*, *b*; Raitanen and Tammela, 1993) and has not been reported for gastric cancer. The aim of the present study was to evaluate the prognostic impact of the Rhesus phenotype in gastric carcinoma patients.

### PATIENTS AND METHODS

For a series of 83 gastric cancer patients undergoing surgical resection according to standard protocols, a panel of potential prognostic parameters was evaluated. Patient-related factors included sex and age. The ABO blood group antigens and the Rhesus D antigen phenotype were serologically determined using standard protocols. Treatment-associated variables considered extent of operation and the presence of residual tumour after surgical treatment. A curative resection (R0, $n$ = 51) is one in which all macroscopically visible tumour has been removed (as determined by the surgeon), and the surgical margins are microscopically tumour free (as determined by the pathologist). Resections with residual microscopic or macroscopic tumour were defined as R1 ($n$ = 10) or R2 ($n$ = 22) respectively. Tumour-associated factors included depth of tumour invasion (pT), involvement of lymph nodes (pN), distant metastasis at time of diagnosis (M) and tumour stage (UICC-stage), classified according to the UICC (International Union against Cancer) recommendations (1992). Tumour localization and tumour diameter were determined and gross appearance of the tumour was evaluated according to the Borrmann classification. Histological tumour type was determined according to the Laurén classification and the degree of tumour cell differentiation (grading) was also assessed (Roder et al, 1993). Lymphatic and blood vessel invasion and the presence of tumour cells in bone marrow, which are proposed as new prognostic factors (Maehara et al, 1995; Jauch et al, 1996), were also considered. Data for these three parameters were not available for all patients.

Prospective follow-up was routinely performed every 6 months and considered tumour recurrence and overall survival. In R0 patients, local and distant tumour relapse were evaluated, while in R1 patients distant metastases only were considered. In patients with macroscopic residual tumour (R2) tumour recurrence was not evaluated. Overall survival data were available for all patients.

Statistical analyses were performed using the Fisher's exact probability test (two-tailed) to examine the association between the Rhesus D phenotype and various clinicopathological and immunohistological parameters. Univariate survival analysis was performed using the Kaplan–Meier method (log-rank test). Multivariate survival analysis was carried out using the Cox proportional hazards regression model (SAS software program, SAS Institute, Cary, NC, USA).

### RESULTS AND DISCUSSION

The Rhesus D-negative phenotype was identified in 18.1% of the total gastric cancer collective. The frequency of the Rhesus D-negative phenotype in the investigated patient cohort is similar to that found in the Caucasian population (Race and Sanger, 1975), suggesting that the Rhesus D-negative phenotype does not predispose to gastric cancer development.

*Received 7 June 1996*
*Revised 24 September 1996*
*Accepted 21 October 1996*

*Correspondence to:* B Mayer

**Table 1** Rhesus factor correlated with clinicopathological factors

| $n^a$ | Parameter | Rhesus factor | | $P^b$ |
|---|---|---|---|---|
| | | Negative | Positive | |
| 83 | Sex | | | |
| | Male | 6 | 39 | 0.26 |
| | Female | 9 | 29 | |
| 83 | Age (median) | | | |
| | ≤ 67 years | 10 | 35 | 0.39 |
| | > 67 years | 5 | 33 | |
| 83 | ABO blood group[c] | | | |
| | O | 6 | 25 | 0.76 |
| | A | 7 | 31 | |
| | B | 2 | 10 | |
| | AB | 0 | 2 | |
| 83 | Residual tumour | | | |
| | R0 | 6 | 45 | 0.039[#] |
| | R1/R2 | 9 | 23 | |
| 83 | Operation[d] | | | |
| | Not extended | 11 | 51 | 0.75 |
| | Extended | 4 | 17 | |
| 83 | pT classification | | | |
| | T1/T2 | 7 | 36 | 0.78 |
| | T3/T4 | 8 | 32 | |
| 83 | pN classification | | | |
| | N0 | 2 | 13 | 0.46 |
| | N1/N2 | 13 | 55 | |
| 83 | M classification | | | |
| | M0 | 8 | 44 | 0.39 |
| | M1 | 7 | 24 | |
| 83 | UICC stage | | | |
| | I/II/IIIA | 6 | 32 | 0.58 |
| | IIIB/IV | 9 | 36 | |
| 83 | Tumour localization | | | |
| | Cardia | 3 | 18 | 0.75 |
| | Other sites | 12 | 50 | |
| 83 | Tumour diameter | | | |
| | ≤ 50 mm | 3 | 25 | 0.25 |
| | > 50 mm | 12 | 43 | |
| 83 | Gross appearance | | | |
| | Borrmann | | | |
| | B1/B2 | 6 | 39 | 0.26 |
| | B3/B4 | 9 | 29 | |
| 83 | Tissue architecture | | | |
| | Laurén | | | |
| | Intestinal | 5 | 36 | 0.25 |
| | Diffuse/mixed | 10 | 32 | |
| 83 | Cellular differentiation | | | |
| | Grading | | | |
| | G1/G2 | 2 | 22 | 0.21 |
| | G3/G4 | 13 | 46 | |
| 73 | Lymphatic vessel invasion* | | | |
| | Absent | 1 | 10 | 0.68 |
| | Present | 12 | 50 | |
| 70 | Blood vessel invasion* | | | |
| | Absent | 4 | 18 | 1.0 |
| | Present | 9 | 39 | |
| 63 | Bone marrow status[e],* | | | |
| | Negative | 7 | 22 | 0.32 |
| | Positive | 4 | 30 | |

[a]$n$, number of investigated patients. [b]$P$, significance according to the exact Fisher's probability test (two-tailed). [c]Blood group A vs blood group O was considered. [d]An extended operation was performed when tumour was infiltrated in neighbouring organs. [e]Patients with a second carcinoma were excluded. *Data were not available for all patients. [#]Significance level $P < 0.05$.

**Figure** Kaplan–Meier survival curves of gastric cancer patients according to the Rhesus D phenotype. (**A**) total patients; Rhesus D-positive ($Rh^+$) patients ($n$ = 68) compared with Rhesus D-negative ($Rh^-$) patients ($n$ = 15), log-rank $P$ = 0.0028. (**B**) R0 patients; Rhesus D-positive patients ($n$ = 45) compared with Rhesus D-negative patients ($n$ = 6), log-rank $P$ = 0.0003

Examination of the relationship between Rhesus factor distribution and a variety of clinicopathological factors revealed a significant correlation only with the presence of residual tumour after surgery (Table 1). The Rhesus D-negative phenotype was detected in only 11.8% (6 out of 51) of the R0 patients, but in 28.1% (9 out of 32) of the patients with macroscopic (R2) or microscopic (R1) residual tumour ($P$ = 0.039). This would appear to reflect an association between the Rhesus D-negative phenotype and advanced disease at time of diagnosis. This relationship was further supported by the fact that all survivors among the R0 patients ($n$ = 17) were Rhesus positive. No significant correlation between Rhesus phenotype and any other clinicopathological factor was observed, neither in the total patient group (Table 1) nor in the R0 patients (data not shown) confirming the results obtained for other tumour types (Kvist et al, 1990; Raitanen and Tammela, 1993; Slater et al, 1993). No correlation was found between the Rhesus phenotype and the expression of the cell adhesion molecules

© Cancer Research Campaign 1997

**Table 2** Clinicopathological factors univariately and multivariately influencing survival of the total patient group and the R0 patient group

| Parameter[a] | Univariate | Multivariate | | |
|---|---|---|---|---|
| | P[b] | P[c] | Relative risk | 95% interval[d] |
| *Total patient group* | | | | |
| Age | 0.033 | NS | – | – |
| Rhesus factor | 0.0028 | NS | – | – |
| Residual tumour[e] | 0.0001 | 0.0001 | 3.4 | 1.8–6.4 |
| Operation | 0.0001 | NS | – | – |
| pT classification[e] | 0.0017 | 0.0001 | 3.3 | 1.8–5.8 |
| pN classification[e] | 0.0003 | 0.032 | 2.0 | 1.1–3.6 |
| M classification[e] | 0.0001 | 0.012 | 2.3 | 1.2–4.4 |
| UICC stage[f] | 0.0001 | NI | – | – |
| Tumour diameter | 0.035 | NS | – | – |
| Gross appearance | 0.015 | NS | – | – |
| Lymphatic vessel invasion[f] | 0.012 | NI | – | – |
| Blood vessel invasion[f] | 0.0004 | NI | – | – |
| *R0 patient group* | | | | |
| Rhesus factor[e] | 0.0003 | 0.0013 | 9.1 | 2.4–34.9 |
| Operation[e] | 0.0001 | 0.0008 | 6.6 | 2.2–19.8 |
| pN classification[e] | 0.012 | 0.0024 | 4.9 | 1.8–13.6 |
| M classification | 0.0034 | NS | – | – |
| UICC stage[f] | 0.002 | NI | – | – |
| Tumour diameter | 0.033 | NS | – | – |

[a]Parameter definitions are given in Table 1. [b]*P*-value was evaluated using the log-rank test. [c]*P*-value was evaluated using the chi-square test. [d]95% interval, 95% confidence interval. [e]Multivariate risk factor (unfavourable vs favourable feature); residual tumour (R1/2 vs R0), pT (T3/4 vs T1/2), pN (N1/2 vs N0), M (M1 vs M0), Rhesus factor (Rh⁻ vs Rh⁺), operation (extended vs not extended, [f]UICC stage, which is reflected by the TNM classification and variables regarding vessel invasion which have missing data were not included in the multivariate analysis. NS, not significant; NI, not included.

E-cadherin and CD44, the immunologically relevant HLA ABC and HLA DR antigens, the accessory molecules ICAM-1 and LFA-3 and the components of the uPA protease system by the primary tumour (data not shown).

Analysis of tumour recurrence and overall survival revealed that the Rhesus D-negative phenotype was associated with poor outcome in both patient cohorts. In the total patient group, local or distant tumour relapse was evaluable in 56 patients (R0, 50 out of 51; R1, 6 out of 10). Tumour recurrence was more frequent in Rhesus D-negative patients (8 out of 9) than in Rhesus D-positive patients (21 out of 47; $P = 0.026$; median follow-up 12 months, range 1–82 months). A similar trend was observed in the R0 group. Tumour recurrence was demonstrable in five out of six Rhesus D-negative patients, but only in 43% (19 out of 44) of the Rhesus D-positive patients ($P = 0.093$, median follow-up 24 months, range 1–82 months). Kaplan–Meier survival curves show that Rhesus D-negative patients had significantly shorter overall survival times than Rhesus D-positive patients in both the total ($n$ = 83) and in the R0 ($n$ = 51) cohort (Figure).

Multivariate analysis was performed to determine if the Rhesus D-negative phenotype is an independent predictor of poor prognosis. This analysis included all parameters listed in Table 1 univariately influencing survival of the patient cohorts at a significance level of $P < 0.05$ (Table 2). In the total patient collective, conventional clinicopathological parameters, i.e. residual tumour after surgical treatment, advanced depth of tumour invasion and the presence of lymph node and distant metastases at time of diagnosis, were found to be independent determinants of poor prognosis (Table 2). The Rhesus factor was not an independent prognostic factor in the total patient cohort. In contrast, in the group of R0 patients who are characterized by a more favourable tumour stage, the Rhesus D-negative phenotype was found to be a powerful independent predictor of poor prognosis ($P = 0.0013$; relative risk 9.1), ranking before the positive nodal status and the performance of an extended operation (Table 2).

An association between Rhesus D-negative phenotype and tumour prognosis has also been observed in other tumours. In oral squamous cell carcinoma, Rhesus D-negative phenotype was identified as an independent parameter associated with tumour progression (Bryne et al, 1991*a*, *b*). In contrast, in colorectal carcinoma Rhesus D-negative phenotype was associated with a favourable tumour stage (Halvorsen, 1986) and in urogenital carcinomas the Rhesus phenotype had no prognostic impact (Kvist et al, 1990, 1992; Raitanen and Tammela, 1993). Interestingly, in small-cell lung cancer, no association was observed between Rhesus phenotype and survival but an increased frequency of Rhesus D-negative individuals was seen, suggesting a predisposal towards this tumour (Cerny et al, 1987, 1992).

The role of the Rhesus factor in tumour progression remains unclear. The function of Rhesus D itself, a molecule that has the structural characteristics of an ion transporter (Telen, 1995), may be important in the maintenance of normal cell growth and differentiation. An alternative possibility is that the Rhesus D-negative phenotype is associated with the loss or alteration in one or more genes directly involved in tumour development. The Rhesus D gene is located on chromosome 1 between p34 and p36 (Cherif-Zahar, 1991), a region recently shown to be the location of a putative gastric carcinoma tumour-suppressor gene (Sano et al, 1991; Ezaki et al, 1996). Inasmuch as the Rhesus D-negative phenotype is usually associated with a deletion of this gene (Hyland et al, 1994), it is possible that this tumour-suppressor gene is also altered or deleted in some Rhesus D-negative individuals, a situation which could directly influence tumour growth and/or tumour progression.

Approximately 50% of curatively (R0) resected gastric cancer patients will die of tumour recurrence despite a favourable tumour stage. In the present study, the Rhesus D-negative phenotype has been detected as a new independent factor of poor prognosis in R0 resected gastric cancer patients. This finding suggests that the routinely evaluated Rhesus phenotype may be an important marker for the selection of R0 resected, high-risk cancer patients who should be considered in prognosis-associated multimodal therapy.

### ACKNOWLEDGEMENTS

The authors thank Mrs C Lorenz and Mr H Spatz for technical assistance, Dr R Babic and Dr MM Heiss for providing the data of the uPA protease system and Dr C Cramer for help in the organization of the follow-up data. This study was supported by the Curt-Bohnewand-Fonds.

### REFERENCES

Bryne M, Eide GE, Lilleng R, Langmark F, Thrane PS and Dabelsteen E (1991*a*) A multivariate study of the prognosis of oral squamous cell carcinomas. *Cancer* **68**: 1994–1998

Bryne M, Thrane PS, Lilleng R and Dabelsteen E (1991*b*) Prognostic value of Rhesus blood groups in oral squamous cell carcinomas. *Cancer* **68**: 2213–2216

© Cancer Research Campaign 1997 

Cerny T, Blair V, Anderson H, Bramwell V and Thatcher N (1987) Pretreatment prognostic factors and scoring system in 407 small-cell lung cancer patients. *Int J Cancer* **36**: 146–149

Cerny T, Fey MF, Oppliger R, Castiglione M, Nachbur B, Gertsch M, Gasser A, Joss RA, Thatcher N, Lind M, Von Rohr A and Nydegger UE (1992) Prevalence of the Rhesus-negative phenotype in Caucasian patients with small-cell lung cancer (SCLC). *Int J Cancer* **54**: 504–506

Cherif-Zahar B (1991) Localization of the human Rh blood group gene structure to chromosome region 1p34.3–1p36 by in situ hybridization. *Hum Genet* **86**: 398–400

Ezaki T, Yanagisawa A, Ohta K, Aiso S, Watanabe M, Hibi T, Kato Y, Nakajima T, Ariyama T, Inazawa J, Nakamura Y and Horii A (1996) Deletion mapping on chromosome 1p in well-differentiated gastric cancer. *Br J Cancer* **73**: 424–428

Halvorsen TB (1986) ABO blood groups, Rhesus types, and colorectal adenocarcinoma. A retrospective study of 747 cases. *Scand J Gastroenterol* **21**: 979–983

Hyland CA, Wolter LC and Saul A (1994) Three unrelated Rh D gene polymorphisms identified among blood donors with Rhesus CCee (r′r′) phenotypes. *Blood* **84**: 321–324

Jauch KW, Heiss MM, Gruetzner U, Funke I, Pantel K, Babic R, Eissner H-J, Riethmüller G and Schildberg FW (1996) The prognostic significance of bone marrow micrometastases in patients with gastric cancer. *J Clin Oncol* **14**: 1810–1817

Kvist E, Krogh J and Rye B (1990) Blood groups and urothelial tumours of the upper urinary tract. *Scand J Urol Nephrol* **24**: 253–255

Kvist E, Krogh J and Hjortberg P (1992) Prognostic variables in patients with prostate cancer: influence of blood group ABO (H), the Rhesus system, age, differentiation, tumour stage and metastases. *Int Urol Nephrol* **24**: 417–423

Maehara Y, Oshiro T, Baba H, Ohno S, Kohnoe S and Sugimachi K (1995) Lymphatic invasion and potential for tumour growth and metastasis in patients with gastric cancer. *Surgery* **117**: 380–385

Race RR and Sanger R (1975) *Blood Groups in Man*, 6th edn. Oxford: Blackwell Scientific Publications

Raitanen MP and Tammela TLJ (1993) Relationship between blood groups and tumor grade, number, size, stage, recurrence and survival in patients with transitional cell carcinoma of the bladder. *Scand J Urol Nephrol* **27**: 343–347

Roder JD, Böttcher K, Siewert JR, Busch R, Hermanek P, Meyer HJ and the German Gastric Carcinoma Study Group (1993) Prognostic factors in gastric carcinoma. Results of the German Carcinoma Study 1992. *Cancer* **72**: 2089–2097

Sano T, Tsujino T, Yoshida K, Nakayama H, Haruma K, Ito H, Nakamura Y, Kajiyama G and Tahara E (1991) Frequent loss of heterozygosity on chromosomes 1q, 5q, and 17p in human gastric carcinomas. *Cancer Res* **51**: 2926–2931

Slater G, Itzkowitz S, Azar S and Aufses JR AH (1993) Clinicopathologic correlations of ABO and Rhesus blood type in colorectal cancer. *Dis Colon Rectum* **36**: 5–7

Tahara E (1995) Genetic alterations in human gastrointestinal cancers. *Cancer* **75**: 1410–1417

Telen MJ (1995) Erythrocyte blood group antigens: not so simple after all. *Blood* **85**: 299–306

© Cancer Research Campaign 1997